# Epidemiological features of domestic and imported cases with COVID-19 between January 2020 and March 2021 in Taiwan

**DOI:** 10.1097/MD.0000000000027360

**Published:** 2021-10-01

**Authors:** Chun-Han Wu, Yu-Ching Chou, Fu-Huang Lin, Chi-Jeng Hsieh, Ding-Chung Wu, Chung-Kan Peng, Chia-Peng Yu

**Affiliations:** a Division of Pulmonary and Critical Care Medicine, Department of Internal Medicine, Tri-Service General Hospital, National Defense Medical Center, Taipei, Taiwan; b School of Public Health, National Defense Medical Center, Taipei, Taiwan; c Department of Health Care Administration, Asia Eastern University of Science and Technology, New Taipei City, Taiwan; d Graduate Institute of Life Sciences, National Defense Medical Center, Taipei, Taiwan.

**Keywords:** COVID-19, domestic, epidemiology, imported, retrospective, SARS-CoV-2

## Abstract

Coronavirus disease-2019 (COVID-19) is a global pandemic affecting numerous countries around the world. This study elaborates Taiwan's epidemiological characteristics from the 2020 to 2021 COVID-19 pandemic from human, temporal, and geographical dimensions. Big data for cases were obtained from a public database from the Taiwan Centers for Disease Control (CDC) in April 2021. The data were analyzed and used to compare differences, correlations, and trends for human, temporal, and geographical characteristics for imported and domestic COVID-19 cases. During the study period, 1030 cases were confirmed and the mortality rate of 1.0%. The epidemiological features indicated that most cases (953/1030, 92.5%) were imported. A comparison of the domestic confirmed and imported cases revealed the following findings: No significant difference of COVID-19 between males and females for sex was observed; For age, the risk of domestic transmission was significantly lower for 20 to 29 years old, higher for 50 to 59 years old, and >60 years old with odds ratios (ORs) (*P* value < .05) of 0.36, 3.37, and 2.50, respectively; For the month of infection, the ORs (*P* value < .05) of domestic confirmed cases during January and February 2020 were 22.428; and in terms of area of residence, the ORs (*P* value < .05) for domestic confirmed cases in northern and southern Taiwan were 4.473 and 0.033, respectively. Thus, the increase in domestic cases may have been caused by international travelers transmitting the virus in March 2020 and December 2020, respectively. Taiwan has been implementing effective screening and quarantine measures at airports. Moreover, Taiwan has implemented and maintained stringent interventions such as large-scale epidemiological investigation, rapid diagnosis, wearing masks, washing hands frequently, safe social distancing, and prompt clinical classifications for severe patients who were given appropriate medical measures. This is the first report comparing imported and domestic cases of COVID-19 from surveillance data from the Taiwan Centers for Disease Control during January 2020 and March 2021. It illustrates that individuals infected during overseas travel are the main risk factors for the spread of COVID-19 in Taiwan. The study also highlights the importance of longitudinal and geographically extended studies in understanding the implications of COVID-19 transmission for Taiwan's population.

## 1 Introduction

In December 2019, clusters of unknown pneumonia were discovered in Wuhan, Hubei Province, China, with most initial cases of the pandemic being related to history of activity at Huanan Seafood Market. China announced on January 7, 2020, that the pathogen was a new type of coronavirus.^[1]^ The pandemic then rapidly spread to other cities and provinces in China as well as around the world, and human-to-human transmission was subsequently verified.^[2,3]^ The World Health Organization announced it as a Public Health Emergency of International Concern on January 30, 2020.^[4]^ The disease caused by the novel coronavirus was referred to as coronavirus disease-2019 (COVID-19) on February 11, 2020,^[5,6]^ and was named severe acute respiratory syndrome coronavirus 2 (SARS-CoV-2) by the International Committee on Taxonomy of Viruses.^[5]^ The novel coronavirus SARS-CoV-2 is a betacoronavirus in the family Coronavirinae (CoV).^[7]^ Key pathogens causing human and animal diseases, CoV is a group of round-shaped, positive-sense single-stranded RNA viruses with a mantle. CoV, named after its viral crown-like spike protein observed under electron microscope, can be divided into alphacoronavirus, betacoronavirus, gammacoronavirus, and deltacoronavirus.^[8]^ Coronaviruses can cause diseases among humans and other vertebrates and are zoonotic.^[9]^ Animal hosts of CoV include bats (the largest population), pigs, cattle, turkeys, cats, dogs, and ferrets, and sporadic cross-species transmission has been reported.^[10]^ Whether the novel coronavirus SARS-CoV-2 that causes COVID-19 has an animal host requires further research and verification.^[11]^ Seven coronaviruses have been known to infect humans, including the alphacoronaviruses HCoV-229E and HCoV-NL63 as well as HCoV-HKU1, HCoV-OC43 or severe acute respiratory syndrome coronavirus, Middle East respiratory syndrome-related coronavirus, and the newly discovered betacoronavirus SARS-CoV-2.^[12]^ Human infection by coronaviruses mainly exhibits respiratory symptoms, including general upper respiratory tract infection symptoms such as nasal congestion, runny nose, cough, and fever. However, patients with severe acute respiratory syndrome coronavirus, Middle East respiratory syndrome-related coronavirus, and SARS-CoV-2 exhibit more severe symptoms than with typical human coronaviruses, with some cases displaying severe pneumonia and respiratory failure.^[13,14]^ Currently, the complete transmission route of SARS-CoV-2 is yet to be fully understood.^[15]^ Investigation of confirmed cases and laboratory tests indicated that droplets, direct or indirect contact with virus-bearing oral and nasal secretions, or long-term close contact (within approximately 2 m) with confirmed patients in a confined space without using respiratory protection increases the risk of human-to-human transmission.^[16]^ Some novel coronaviruses may cause diarrhea in animals, and the virus can be found in their feces.^[17]^ Human feces may also be tested positive for SARS-CoV-2 nucleic acid, in turn leading to spread of the virus among humans.^[18]^ According to information released by the World Health Organization, the incubation period for infection with SARS-CoV-2 to onset of illness is 1 to 14 days, and a confirmed patient may be infectious 2 days before the onset of illness.^[19]^ The clinical manifestations of currently known confirmed COVID-19 cases include fever, dry cough, fatigue, and shortness of breath. Other symptoms include muscle pain, headache, sore throat, and diarrhea, and some cases display loss (or abnormality) of smell or taste.^[20]^ According to current epidemiological information, most patients can recover, whereas a small number progress to severe pneumonia, respiratory distress syndrome, multiple organ failure, shock, or even death.^[21]^ Most deaths have underlying medical history such as diabetes, chronic liver disease, renal insufficiency, or cardiovascular disease.^[22,23]^

Taiwan, located at 23°4′ north latitude and 121°0′ east longitude, has a subtropical climate, with monthly average temperatures and monthly average relative humidity of 16°C to 29°C and 75% to 90%, respectively. A developed country, Taiwan's per capita gross domestic product is US$27,171.^[24]^ Despite the large number of imported COVID-19 cases (and a number of domestic cases), the use of big data in epidemiological information to explore COVID-19-related risks in Taiwan has been sparse. Thus, the purpose of this study was to employ the Taiwan National Infectious Disease Statistics System (TNIDSS) to explore the epidemiological characteristics, differences, and trends regarding sex, age, season, and residential area of confirmed imported and domestic COVID-19 cases in Taiwan from January 2020 to March 2021.

## 2 Materials and methods

### 2.1 Ethical statement

This study did not require ethical approval because it used information freely available in the public domain and the analysis of open-source datasets in which the data were properly anonymized.^[25,26]^

### 2.2 Definition of reported and confirmed cases^[27]^

1. Definition of clinical conditions

Patient displaying any of the following conditions: (1-1) fever (≥38°C) or respiratory tract symptoms; (1-2) abnormal smell and taste or diarrhea of unknown cause; or (1-3) clinical symptoms highly suspected by physicians to be community-acquired pneumonia.

2. Definition of inspection conditions

Clinical specimens (e.g., nasopharyngeal or throat swab, sputum, or lower respiratory tract extracts) that are tested positive in the novel coronavirus molecular biology nucleic acid test—identification of the novel coronavirus.

3. Definition of epidemiological conditions

Individuals with any of the following conditions within 14 days before the onset of illness: (3-1) a history of foreign travel or residence, or contact with individuals from abroad who have fever or respiratory symptoms; (3-2) close contact with highly probable or confirmed cases with symptoms consistent with COVID-19 (including providing care, staying with them in the same space without proper protection, or having direct contact with their respiratory tract secretions or body fluids); and (3-3) cluster infection phenomenon.

4. Definition of reported case

Individuals who meet any of the conditions stated in (4-1) through (4-4): (4-1) “meet any of the clinical conditions in (1-1) and epidemiological conditions”; (4-2) “meet clinical conditions (1-2) and epidemiological conditions (3-1) or (3-2)”; (4-3) “meet clinical condition (1-3)”; or (4-4) “meet the inspection conditions.”

5. A confirm case is defined as a patient who must meet the test conditions.

### 2.3 Data source

Taiwan has a population density of 627 per km^2^, an area of 36,188 km^2^, and a population of approximately 23.5 million. The majority (95%) of the population lives in western Taiwan, which is divided into northern, central, and southern regions. Only 5% of Taiwan's population lives in eastern Taiwan, and this population is categorized as disadvantaged based on medical care and socioeconomic status. In addition, Taiwan has 3 outlying islands: Penghu, Kinmen, and Matsu islands.

This study used TNIDSS, which is the public database of the online platform of Taiwan Centers for Disease Control (CDC).^[28]^ The database includes all Category 1 to 5 communicable diseases as stipulated by the Communicable Disease Control Act. Since January 2020, Taiwan CDC has made available information on confirmed COVID-19 cases in the TNIDSS, providing the public, academics, and media with current information on the COVID-19 pandemic. In addition, Taiwan announced in January 2020 that COVID-19 is a Category 5 communicable disease under the Communicable Disease Act, and the data available for inquiry include the number of domestic and imported COVID-19 cases from January 2020 to March 2021.

The internet datasets contain information on gender, age, month of case confirmation, and area of residence of those with COVID-19. The datasets do not contain information regarding the medical history of patients, signs and symptoms, or treatment.

### 2.4 Statistical analyses

The number of domestic and imported COVID-19 cases from January 2020 to March 2021 was determined using the database, and the distribution of their epidemiological characteristics (sex, age, year and month of confirmed illness, season, and area of residence), differences, and results were examined. Descriptive data are shown as mean and summary statistics, where appropriate. Categorical variables were compared using the chi-square/Fisher exact probability test. The logistic regression analysis was used to determine the risk of COVID-19, the results were presented as an odds ratios (ORs) with a 95% confidence interval. All statistical analyses were performed using SPSS software (IBM SPSS Statistics 21; Asia Analytics Taiwan Ltd., Taipei, Taiwan). All statistical tests were 2-sided with an α level of 0.05. Values of *P* < .05 were considered statistically significant.

## 3 Results

**Figure 1:**
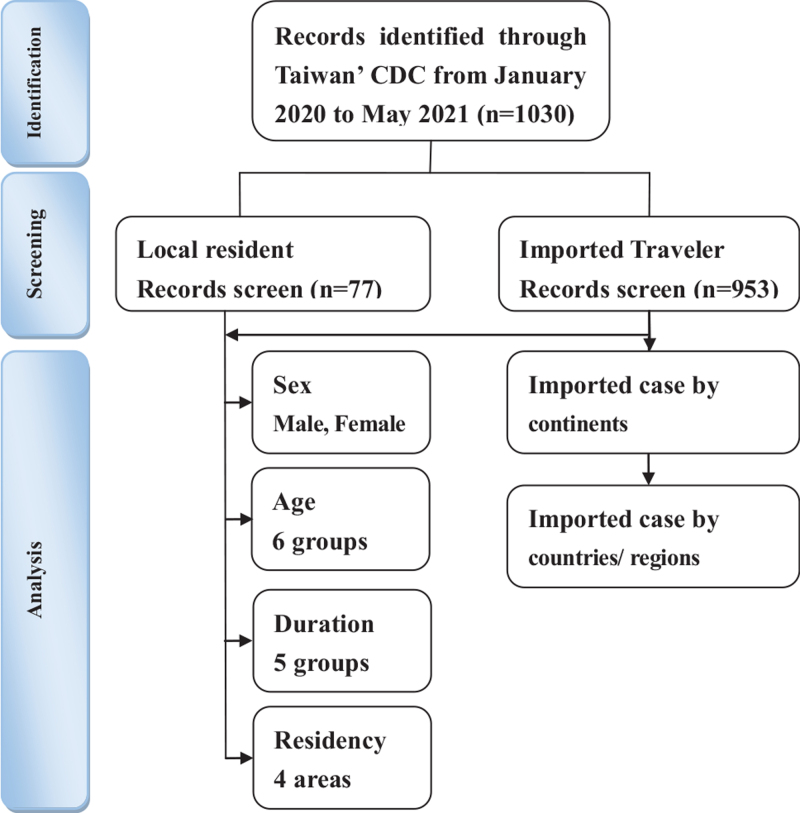
The flowchart of study sample selection from Taiwan Centers for Disease Control Database in Taiwan during January 2020 and March 2021.

A total of 1030 patients were identified in the Taiwan's CDC database from January 2020 to March 2021. A detailed flowchart is shown in Figure 1. Confirmed COVID-19 cases were 1030, for which data related to risk of infection (sex, age, month of infection, and area of residence) with their statistical significances that were obtained (Table 1). A comparison of the domestic confirmed and imported cases revealed the following findings: No significant difference of COVID-19 cases between males and females for sex was observed; For age, the risk of domestic transmission was significant lower for 20 to 29 years old, higher for 50 to 59 years old, and >60 years old with ORs (*P* value < .05) of 0.36, 3.37, and 2.50, respectively; For the month of infection, the ORs (*P* value < .05) of domestic confirmed cases during January and February 2020 were 22.428; and in terms of area of residence, the ORs (*P* value < .05) of domestic confirmed cases in northern and southern Taiwan were 4.473 and 0.033, respectively (Table 1).

**Table 1:** Distribution of confirmed COVID-19 cases compared between importation cases and domestic transmission by sex, age, duration and residential area.

Variables	All cases N = 1030	Local resident N = 77	Imported traveler group N = 953	*P*	*OR*	*95%CI*
Sex						
Male	522	33	489	.153	–	–
Female	508	44	464	.153	–	–
Age group						
<19	50	5	45	.487	–	–
20–29	400	15	385	<.001	0.357	0.200–0.637
30–39	264	14	250	.120	–	–
40–49	120	10	110	.704	–	–
50–59	97	18	79	<.001	3.375	1.898–6.003
>60	99	15	84	.003	2.503	1.364–4.592
Duration						
2020/1–2020/2	45	25	20	<.001	22.428	11.697–43.002
2020/3–2020/5	400	30	370	.981	–	–
2020/6–2020/8	52	0	52	–	–	–
2020/9–2020/11	196	0	196	–	–	–
2020/12–2021/3	337	22	315	.420	–	–
Resident area						
Northern area	612	66	546	<.001	4.473	2.332–8.577
Central area	140	10	130	.872	–	–
Southern area	270	1	269	.001	0.033	0.005–0.242
Eastern area	8	0	8	–	–	–

CI = confidence interval, COVID-19 = coronavirus disease-2019, OR = odds ratio.

During the survey period of January 2020 to March 2021, the total number of confirmed COVID-19 cases was 1030 (including 77 domestic confirmed cases and 953 imported cases). Among the 953 imported cases, 47.0% were acquired in Asia, 23.9% were acquired in Europe, 20.0% were acquired in America, 4.2 were acquired in Africa, 0.5% were acquired in Oceania, and 4.3% were acquired in another country/region (Table 2).

**Table 2:** Confirmed COVID-19 cases imported to Taiwan between January 2020 and March 2021 by continent.

Region	All cases (%) N = 953	2020/1–2020/12 (%) N = 764	2021/1–2021/3 (%) N = 189
Asia	448 (47.0)	336 (44.0)	112 (59.3)
Europe	228 (23.9)	207 (27.1)	21 (11.1)
Americas	191 (20.0)	149 (19.5)	42 (22.2)
Africa	40 (4.2)	26 (3.4)	14 (7.4)
Oceania	5 (0.5)	5 (0.6)	0
Others	41 (4.3)	41 (5.4)	0

COVID-19 = coronavirus disease-2019.

A total of 10 deaths (4 domestic cases and 6 imported cases; 8 men and 2 women; aged 40–80) from COVID-19 were recorded during the study period (Table 3).

**Table 3:** Characteristics of cases died from COVID-19 from January 2020 to March 2021 reported in Taiwan's CDC surveillance data.

Death cases no.^∗^	Confirmed day	Death day	Domestic or imported	Transmission route	Gender	Age
1	2020/2/15	2020/2/15	Domestic	Contact infections	Male	61–70
2	2020/2/23	2020/3/20	Domestic	Family cluster	Male	80–90
3	2020/2/28	2020/3/29	Domestic	Nosocomial infections	Female	50–60
4	2020/3/19	2020/3/29	Imported	Returning from Austria and Czech Republic	Male	40–50
5	2020/3/23	2020/3/29	Imported	Returning from Spain	Male	60–70
6	2020/3/19	2020/4/9	Imported	Returning from Egypt	Male	70–80
7	2020/3/24	2020/5/11	Imported	Returning from USA	Male	40–50
8	2021/1/29	2021/1/29	Domestic	Nosocomial infections	Female	80–90
9	2020/1/31	2021/2/3	Imported	Returning from United Kingdom	Male	70–80
10	2020/12/29	2021/3/5	Imported	Returning from Myanmar	Male	60–70

CDC = Centers for Disease Control, COVID-19 = coronavirus disease-2019.

∗ The serial number is based on the date of death.

**Figure 2:**
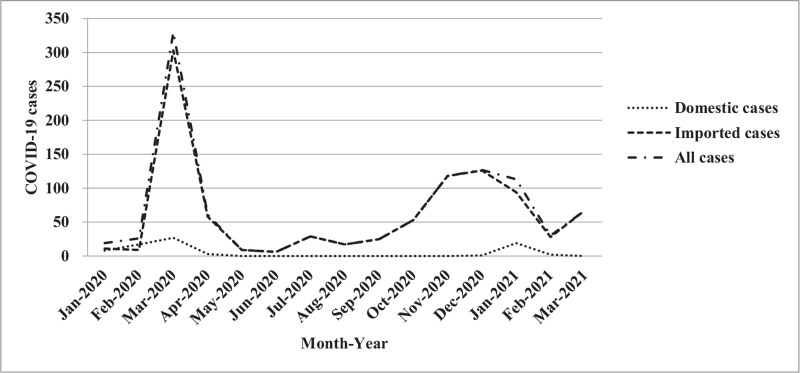
Monthly distribution of COVID-19 cases estimated for domestic transmission, importation, and all cases in Taiwan. COVID-19 = coronavirus disease-2019.

**Figure 3:**
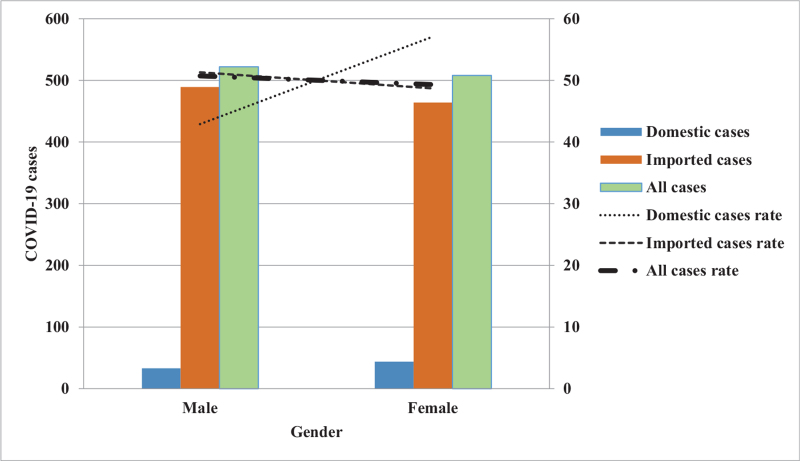
Sex-specific COVID-19 cases estimated for domestic transmission, importation, and all cases in Taiwan. COVID-19 = coronavirus disease-2019.

**Figure 4:**
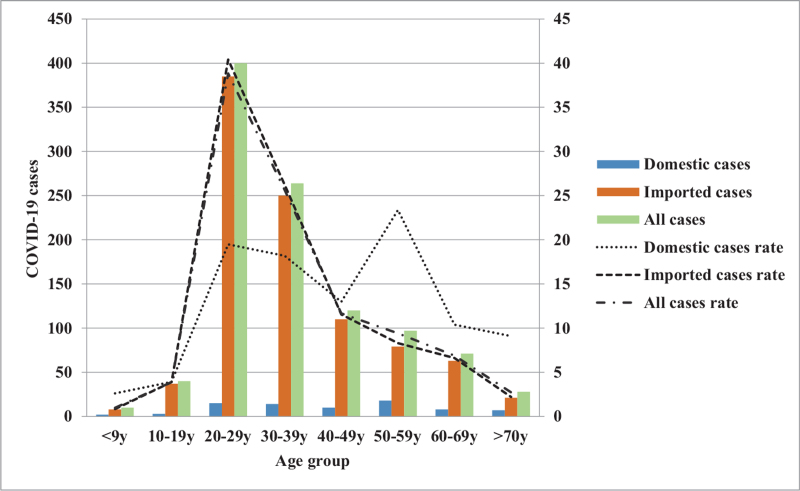
Age-specific COVID-19 cases estimated for domestic transmission, importation, and all cases in Taiwan. COVID-19 = coronavirus disease-2019.

**Figure 5:**
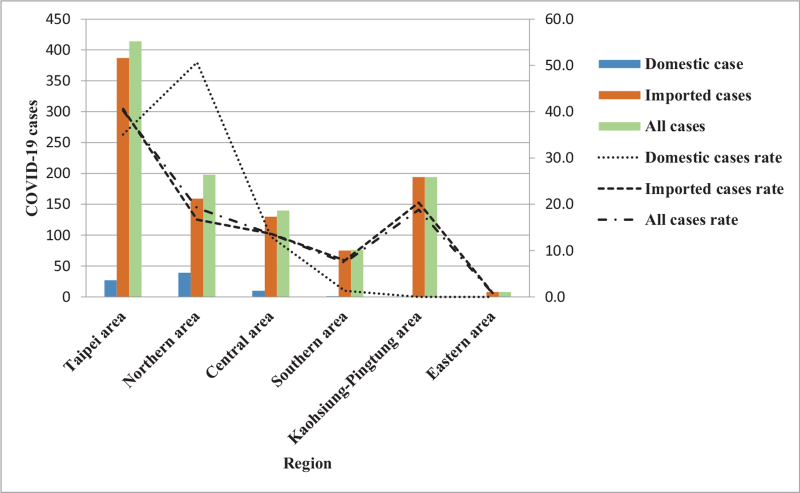
COVID-19 cases in 6 geographic regions estimated for domestic transmission, importation, and all cases in Taiwan. COVID-19 = coronavirus disease-2019.

Furthermore, the trends and impacts of COVID-19 based on an epidemic curve composed of “imported or domestic patients’ sex difference, age group difference, changes in monthly incidence, and different geographic regions” were analyzed.^[28]^ For trends as to the monthly number of imported and domestic cases from January 2020 to March 2021. The number of imported cases peaked along the epidemic curve in March and, again, in December 2020, with the number of cases being 303 and 126, respectively; while the domestic cases also exhibited an epidemic curve peak in March 2020 and then again in January 2021 with the number of cases being 27 and 19, respectively (Fig. 2). By analyzing the changes in the trends for all patients based on gender showed that the cases number for imported cases was 51.3% (489/953) for men and 48.7% (464/953) for women; while the epidemic curve distribution of domestic cases was 42.9% (33/77) for men, 57.1% (44/77) for women (Fig. 3). An examination of these trends changes with age distribution as there is a peak along the epidemic curve for imported cases for patients between 20 and 29 years old, which accounts for 40.4% (385/953) of the total number of imported cases. Further, while there is a peak along the epidemic curve for domestic cases for patients between 20 and 29 years old and 50 and 59 years of age and accounted for 19.5% (15/77) and 23.4% (18/77) of domestic cases, respectively (Fig. 4). An analysis of these trends indicated changes in the distribution across all residential areas. Further, there was an epidemic curve peak for those imported cases living in Taipei and Gao-Ping; and the imported case rate was 40.6% (387/953) and 20.4% (194/953), respectively; while for domestic cases in northern Taiwan had a peak along the epidemic curve, which accounted for 50.6% (39/77) of domestic cases (Fig. 5).

**Figure 6:**
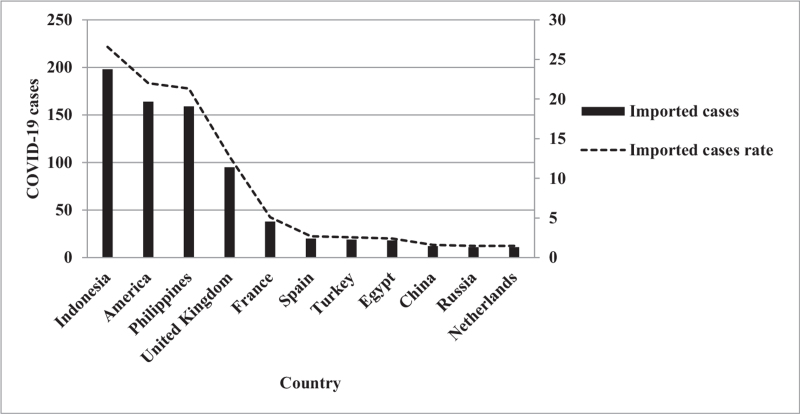
Imported COVID-19 cases in Taiwan by the importation country. COVID-19 = coronavirus disease-2019.

Among the 953 imported cases, the top 10 countries were Indonesia (26.6%, 198/745), the United States (22.0%, 164/745), the Philippines (21.3%, 159/745), the United Kingdom (12.8%, 95/745), France (5.1%, 38), Spain (2.7%, 20/745), Turkey (2.6%, 19/745), Egypt (2.4%, 18/745), China (1.6%, 12/745), Russia (1.5%, 11/745), and the Netherlands (1.5%, 11/745) (Fig. 6).

## 4 Discussion

Due to transport convenience, travelers may be exposed to infectious diseases or become ill overseas and may spread the disease from 1 country to another, causing transnational pandemics. Thus, international travelers are of great significance in the epidemiology of infectious diseases.^[29]^ For example, in the past decade, international travelers have faced threats from diseases such as Ebola, Zika, and chikungunya. Infectious disease surveillance networks are constantly evolving due to time and environmental changes (e.g., the Global TravEpiNet, an alliance formed by health clinics across the United States, and Taiwan CDC's travelers’ health surveillance network) that have further contributed to meeting a growing demand for international traveler surveillance. In addition, the risks of travel-related diseases vary by destination and traveler characteristics. To assess the actual risks for international travelers, this study collected and analyzed correlations between imported cases of COVID-19 (i.e., international travelers) at a specific period and domestic cases in that same period.

Due to the explosive growth of international travel, the diverse networks and complexities of human mobility have become essential factors that gave rise to the rapid spread of COVID-19 globally in 2020. A Korean study reported^[30]^ that most of COVID-19 cases imported to Korea were from other Asian countries (50.6%), followed by America (28.1%), and Europe (17.8%). This result is similar to that obtained in this study. Another study,^[31]^ further indicated that the majority of cases came from Asia (41.4%) and Europe (26.5%); specifically from the United Kingdom, Bangladesh, the United States, the Philippines, and France that represented 64.9% of the total imported international cases. This result is also similar to that obtained in this research because COVID-19 cases were dramatically expanding and increasing in the most countries from March 2020 until March 2021. Therefore, this study suggests that the diverse network and complexity of human mobility has become an essential risk factor that gave rise to and continued the spread of COVID-19 globally within a short period of time.

Taiwan is one of a few countries that had experienced an early stage of the COVID-19 pandemic. Based on the chronological order of COVID-19 infections, the results indicated that the increased numbers of domestic cases may have been caused by international travelers who were transmitting SARS-CoV-2 in Taiwan in January 2020 and March 2021. Despite recent achievements in vaccines, anti-viral drugs, and medical infrastructure, when faced with imported cases from visitors to Taiwan, the country implemented an effective screening and quarantine procedures at airports. In particular, Taiwan has implemented and maintained stringent interventions such as large-scale epidemiological investigations, rapid diagnosis, wearing masks, washing hands frequently, safe social distancing, and prompt clinical classification for severe patients with appropriate medical measures taken quickly. The number of overseas travelers seriously affects the continued occurrences of local cases in Taiwan. Based on this relationship, we have assessed the epidemiological risk factors for the spread of COVID-19 has expanded from January 2020 to March 2021 in Taiwan.

According to studies elsewhere, most confirmed COVID-19 cases are concentrated in metropolitan areas,^[32]^ which concurs with the findings of this study. This study revealed that with the concentration of imported cases in metropolitan areas of northern Taiwan (particularly Taipei City), domestic cases also rose significantly, resulting in increased risks for local residents. Moreover, this study discovered that patients with COVID-19 were concentrated in the densely populated metropolitan areas of Taipei City and New Taipei City in northern Taiwan (38%, 391/1030), which was inferred to be related to the characteristics of COVID-19 being transmitted rapidly in crowded and confined spaces.^[33]^ This study inferred that the incidence rate and spreading of COVID-19 is geographically concentrated and limited. Accordingly, the SARS-CoV-2 infections imported through overseas travel are likely to be the major risk factor for the COVID-19 pandemic in Taiwan. These findings may serve as the basis for governments or public health experts to formulate pandemic prevention policies and health resource allocation decisions.

Furthermore, this study determined that the risks for local residents being infected with COVID-19 differed according to the month of infection and place of residence. Another possible reason is the disproportionate attention to pandemic prevention by patients with COVID-19 in different months of infection and the varying effectiveness of pandemic prevention in different areas of residence, which led to the rise in the risks for COVID-19 infections. The aforementioned results can serve as a reference for the Taiwanese government in formulating pandemic prevention policies, preventive medicine, and clinical care.

According to the literature, no differences were observed in the sex of patients with COVID-19,^[34]^ which was consistent with the results from the number of confirmed COVID-19 cases (imported and domestic cases) in this study. According to another study,^[35]^ patients with COVID-19 are mainly adults, and most cases of childhood infections were related to contact with family members individually or at family gatherings involving adults with confirmed infections. The results of this study have revealed that the majority of patients with COVID-19 in Taiwan are adults (aged 20 years of age or older, 95.1%, 980/1030) and that the source of childhood infection was family gatherings, similar to the findings of the aforementioned study. Moreover, the majority of imported cases fell within the age group of 20 to 29 years of age, which was similar to findings reported elsewhere^[36]^; however, the age group of 50 to 59 years of age had the highest proportion of domestic cases. This indicates that young people infected with COVID-19 may spread the disease to Taiwan through international travel, leading to a sharp rise in the infection rate among low-immunity populations (middle-aged and older adults) in the destination country (Taiwan), which in turn causes a health threat and creates a health care burden. Regarding the epidemiological trends of COVID-19, the results indicated that when compared with other age groups, patients aged 20 to 39 years (including imported and domestic cases) accounted for the highest proportion (percentage) regardless of sex, age, or area of residence, which concurs with findings reported elsewhere.^[35]^ The possible reason is that young people have milder COVID-19 symptoms and higher infectivity; thus, the number of confirmed cases for this group was higher.

According to these findings, the mortality of patients with COVID-19 in Taiwan was 1.0%; men accounted for most of these deaths (80%, 8/10). Deaths were also concentrated in the age group of 40 to 80 years. These findings concurred with those of other studies.^[37–39]^ Most COVID-19 cases involving children who exhibited mild symptoms and with sporadic deaths^[40]^; no children died in Taiwan. A noteworthy aspect is that the sources of infection for patients with COVID-19 in Taiwan (including those resulting in death) were contact infection, family gatherings, and hospital infection; no community spread was recorded. A possible reason is the decisive and accurate pandemic prevention policies by the government and the joint efforts of the public and the government to combat the pandemic.

## 5 Limitation

Taiwan's epidemiological characteristics for COVID-19 in human, temporal, and geographical dimensions are recommended to be incorporated into the planning and strategy implementation of public health measures and pandemic prevention worldwide. This study has the following limitations: the infectious disease data published by the Taiwan CDC on the internet platform provided only basic epidemiological data of patients with COVID-19; they do not contain clinical data or detailed experimental procedures; however, the authenticity of the positive confirmed cases announced by Taiwan's CDC was convincing and differences or trends in patient clinical data or symptoms could not be compared; and the data disclosed on the platform also do not contain any information related to the SARS-CoV-2 genotype. Thus, the study was incapable of determining: the genotype of SARS-CoV-2 currently prevalent in Taiwan; and/or the genetic relationship between the SARS-CoV-2 genotypes in Taiwan and other countries. However, this study had 1advantage, which is the instant and accurate data disclosed by Taiwan's public sector on an online public platform. In addition, the platform has maintained an abundance of information over a long period, enabling researchers and institutions to describe or employ statistical methods on the monitored infectious disease data to produce academic value, promote effective disease control, and maintain the health of the Taiwanese public.

## 6 Conclusions

In conclusion, this study was the first to report on the human, temporal, and geographic characteristics and trends for imported and domestic COVID-19 cases in Taiwan from January 2020 to March 2021. It verified that an increase in the number of international travelers infected with COVID-19 within a short period of time in Taiwan resulted in increased domestic cases. This linkage and the relationship of the disease did indeed burdened Taiwan's medical staff and threaten people's health. Moreover, most of the imported cases were young people; however, the domestic cases were mostly middle-aged and older adults. The low-immunity populations in the visited country were also burdened. The increase in the number of visitors infected with COVID-19, the visiting younger population, and the places of residence of the visiting individuals were all verified risk factors. Moreover, most of the confirmed cases were concentrated in northern Taiwan.

## 7 Future direction

This information will be useful for policymakers and clinicians directing prevention and control activities for COVID-19, a severe illness and an enormous burden on Taiwan. The identified data may inform future surveillance and research efforts in Taiwan to minimize the impact of future COVID-19 outbreaks as well as other unknown future pandemics.

## Acknowledgment

This manuscript was edited by Academic Editing USA.

## Author contributions

**Conceptualization:** Chung-Kan Peng, Chia-Peng Yu.

**Data curation:** Chia-Peng Yu.

**Formal analysis:** Chia-Peng Yu.

**Investigation:** Fu-Huang Lin, Chi-Jeng Hsieh, Ding-Chung Wu.

**Methodology:** Chung-Kan Peng, Chia-Peng Yu.

**Validation:** Chun-Han Wu, Yu-Ching Chou, Fu-Huang Lin, Chi-Jeng Hsieh, Ding-Chung Wu, Chung-Kan Peng, Chia-Peng Yu.

**Writing – original draft:** Chun-Han Wu, Chia-Peng Yu.

**Writing – review & editing:** Yu-Ching Chou, Chung-Kan Peng, Chia-Peng Yu.
